# Suppression of 3*C*-Inclusion Formation during Growth of 4*H*-SiC Si-Face Homoepitaxial Layers with a 1° Off-Angle

**DOI:** 10.3390/ma7107010

**Published:** 2014-10-17

**Authors:** Keiko Masumoto, Hirokuni Asamizu, Kentaro Tamura, Chiaki Kudou, Johji Nishio, Kazutoshi Kojima, Toshiyuki Ohno, Hajime Okumura

**Affiliations:** 1 R & D Partnership for Future Power Electronics Technology, 16-1 Onogawa, Tsukuba, Ibaraki 305-8569, Japan; E-Mails: h-asamizu@fupet.or.jp (H.A.); k-tamura@fupet.or.jp (K.T.); c-kudo@fupet.or.jp (C.K.); j-nishio@fupet.or.jp (J.N.); kazu-kojima@aist.go.jp (K.K.); t-ono@fupet.or.jp (T.O.); h-okumura@aist.go.jp (H.O.); 2 National Institute of Advanced Industrial Science and Technology, Central 2 1-1-1 Umezono, Tsukuba, Ibaraki 305-8568, Japan; 3 ROHM Co., Ltd., 21 Saiin Mizosaki-cho, Ukyo-ku, Kyoto 615-8585, Japan; 4 Panasonic Corporation, 800 Higashiyama, Uozu, Toyama 937-8585, Japan; 5 Toshiba Corporation, 1 Komukai-Toshiba-cho, Saiwai, Kawasaki, Kanagawa 212-8582, Japan; 6 Hitachi, Ltd., 1-280 Higashi-koigakubo, Kokubunji-shi, Tokyo 185-8601, Japan

**Keywords:** silicon carbide, epitaxial growth, low off-angle, 3*C* inclusion, *in situ* etching, C/Si ratio

## Abstract

We grew epitaxial layers on 4*H*-silicon carbide (SiC) Si-face substrates with a 1° off-angle. The suppression of 3*C*-inclusion formation during growth at a high C/Si ratio was investigated, because a growth technique with a high C/Si ratio is needed to decrease residual nitrogen incorporation. 3*C* inclusions were generated both at the interface between the substrate and epitaxial layer, and during epitaxial growth. 3*C*-SiC nucleation is proposed to trigger the formation of 3*C* inclusions. We suppressed 3*C*-inclusion formation by performing deep *in situ* etching and using a high C/Si ratio, which removed substrate surface damage and improved the 4*H*-SiC stability, respectively. The as-grown epitaxial layers had rough surfaces because of step bunching due to the deep *in situ* etching, but the rough surface became smooth after chemical mechanical polishing treatment. These techniques allow the growth of epitaxial layers with 1° off-angles for a wide range of doping concentrations.

## 1. Introduction

Silicon carbide (SiC) is expected to be widely applied in the power devices used to control various high-voltage functions, because the power conversion efficiency of SiC devices is higher than that of silicon ones. In particular, among the many polytypes such as 4*H*, 6*H* and 3*C*, 4*H*-SiC is mainly used for developing SiC power devices because of its higher electron mobility and wider bandgap.

Homoepitaxial growth of 4*H*-SiC layers is essential for controlling the doping concentration to produce functional 4*H*-SiC devices. This is because SiC boules are usually grown by sublimation, and it is difficult to control the doping concentration using this technique. Unfortunately, in homoepitaxial growth of 4*H*-SiC, other-polytype inclusions are easily generated because of their low formation energy [1]. However, 4*H*-SiC homoepitaxial growth without other-polytype inclusions has been achieved by using substrates with several off-angles [2,3]. This technique is called “step-controlled epitaxial growth” [3]. Now, 4*H*-SiC epitaxial growth on substrates with a 4° off-angle is standard for fabricating SiC devices.

Recently, lowering the off-angle below 4° has been attempted because it offers several advantages [4,5,6]. First, it was reported that lowering the off-angle is an effective way to suppress the anisotropy due to the large off-angles of SiC trench metal-oxide-semiconductor field-effect transistors (MOSFETs) [7]. Trench MOSFETs are well known to have low on-resistances due to the high cell density, but the anisotropic channel properties on the trench sidewalls restrict the cell structure [7]. In addition, the basal plane dislocation (BPD) density decreases with decreasing the off-angle [8,9]. In the past time, we found that an epitaxial layer with a 1° off-angle has low BPD density of 0.2 cm^−2^ [8]. The number of BPDs in epitaxial layers should be reduced, because they cause stacking faults that increase the forward voltage in bipolar devices [10]. BPDs in substrates convert to threading edge dislocations in epitaxial layers, and the conversion ratio can be increased by lowering the off-angle [11]. Finally, it is obvious that lowering the off-angle reduces the wafer cost, because the amount of waste generated by cutting wafers on a diagonal line can be reduced. This advantage becomes considerably important if large-diameter wafers are used. Indeed, there are many reports in 6-inch SiC wafers, and the mainstream size will be 6 inches in the near future [12,13,14].

In the past, we have found that the step-controlled epitaxial growth on 4*H*-SiC Si-face substrates can be achieved when the off-angle is reduced down to 0.8° [5]. However, a low C/Si ratio has been needed for such growth in order to suppress inclusions of other polytypes [6]. This is problematic, because the background carrier concentration increases with decreasing C/Si ratio as the residual nitrogen incorporation increases as a result of the site-competition effect [15]. For example, growth at a C/Si ratio of less than 0.7 is needed to suppress other polytype inclusions, but the background carrier concentration is 5.8 × 10^15^ cm^–3^ under these conditions [6]. This background carrier concentration value is high because the drift layer carrier concentration of 3.3 kV MOSFETs, for example, is around 3 × 10^15^ cm^–3^. For this reason, a growth technique with a high C/Si ratio is necessary in order to control the doping concentration in a wide range.

In this study, we grew epitaxial layers on 4*H*-SiC Si-face substrates with a 1° off-angle and investigated the formation of other-polytype inclusions with the goal of suppressing these defects even for growth at a high C/Si ratio.

## 2. Results and Discussion

### 2.1. Defects Generated in 4H-SiC Si-Face Epitaxial Layers with a 1° Off-Angle

**Figure 1:**
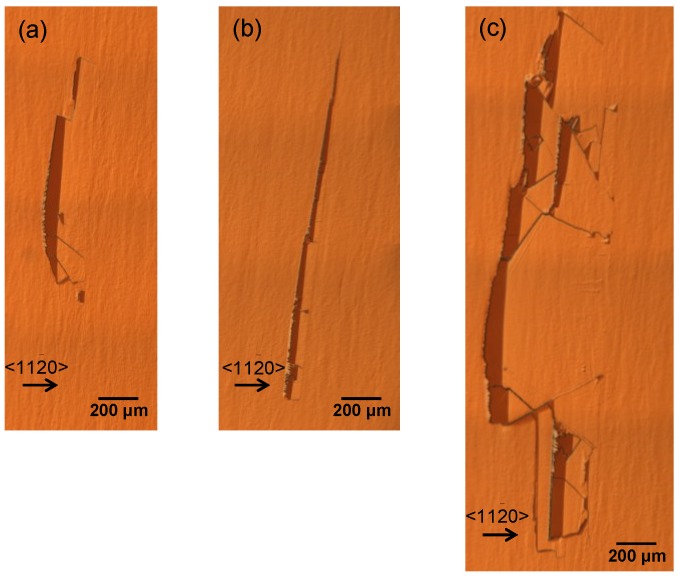
(**a**–**c**) Nomarski optical microscope (NOM) images of surface defects generated in 4*H*-SiC Si-face epitaxial layers with a 1° off-angle.

Figure 1a–c shows Nomarski optical microscope (NOM) images of the surface defects generated in this experiment. These surface defects have various shapes such as trapezoidal shapes, line shapes and so on. We measured the length along the $<\bar{1}100>$ and $<11\bar{2}0>$ directions of 10 surface defects generated in a 9.4 μm thick epitaxial layer from the NOM images. The length along the $<\bar{1}100>$ direction was from 1.2 mm to 6.5 mm, which suggests that these surface defects negatively affect the usable area of epitaxial layers because of their large size. Moreover, the length along the $<11\bar{2}0>$ direction was from 83 μm to 540 μm. We can estimate the points at which the surface defects were generated by multiplying tangent 1° by the length along the $<11\bar{2}0>$ direction. When the length along the $<11\bar{2}0>$ is about 540 μm, the surface defects are supposed to generate at the interface between the substrate and epitaxial layer. Therefore, the defects with length of 540 μm were generated at the interface between the substrate and epitaxial layer, and the defects with lengths less than 540 μm were generated during epitaxial growth. It is concluded that defect generation occurred both at the interface between the substrate and epitaxial layer, and during epitaxial growth.

**Figure 2:**
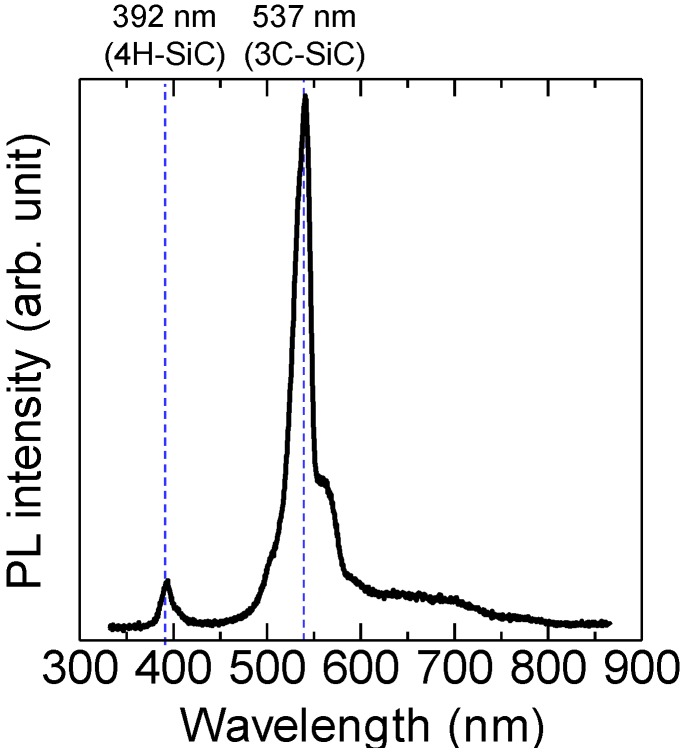
Photoluminescence (PL) spectrum recorded on the surface defect generated in the 4*H*-SiC Si-face epitaxial layers with a 1° off-angle.

Figure 2 shows a photoluminescence (PL) spectrum recorded on a surface defect. It emits luminescence at 392 nm and 537 nm, which correspond to the luminescence of 4*H*-SiC and 3*C*-SiC, respectively [16]. The surface defect has 4*H*- and 3*C*-SiC parts. This indicates that the surface defect is a 3*C* inclusion. We found that all of the defects generated in this experiment were 3*C* inclusions by investigating their luminescence properties.

**Figure 3:**
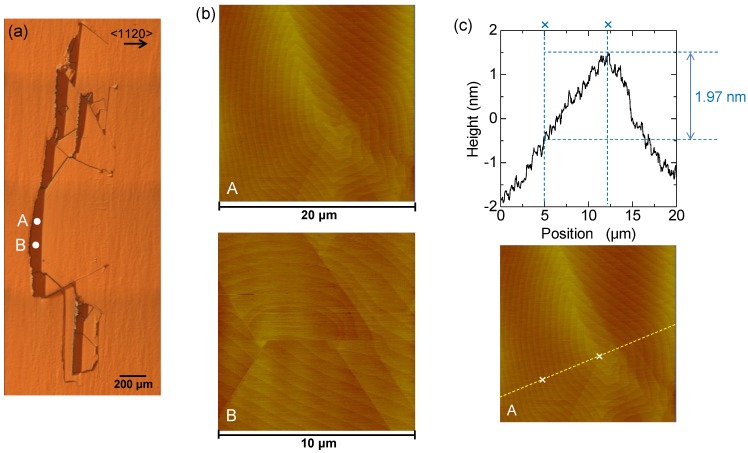
(**a**) NOM image showing atomic force microscopy (AFM) measuring positions (labeled A and B); (**b**) corresponding AFM images; (**c**) cross-sectional profile of the AFM image (yellow dashed line at position A).

We investigated the surface morphology of the 3*C* inclusion shown in Figure 1c around its presumed original position by atomic force microscopy (AFM) to determine the trigger of 3*C*-inclusion formation. Figure 3a presents a NOM image showing the AFM measuring positions (labeled A and B) in the 3*C*-SiC region. Figure 3b shows the corresponding AFM images, which show steps not along the substrate steps, *i.e.*, not along the $<11\bar{2}0>$ direction. The center of a nucleus pattern can be seen at around the center of these figures. It is thought that 3*C*-SiC nucleation occurs because positions A and B are in the 3*C*-SiC region. This indicates that 3*C*-SiC nucleation causes the formation of 3*C* inclusions. Figure 3c shows the cross-sectional profile of the AFM image (yellow dashed line at position A). The height of the nine-layer spiral growth hillock between the two cross marks is 1.97 nm. The height of one layer is 0.22 nm, which is approximately equal to the single Si-C bilayer height of 0.25 nm.

Thus, the generation of variously shaped 3*C* inclusions is triggered by 3*C*-SiC nucleation generated both at the interface between the substrate and epitaxial layer, and during the epitaxial growth of 4*H*-SiC Si-face epitaxial layers with a 1° off-angle. 

### 2.2. Suppression of 3C Inclusions

We first tried to suppress the generation of 3*C* inclusions at the interface between the substrate and epitaxial layer. The relationship between the 3*C*-inclusion density and the *in situ* etching depth was investigated, because it has been reported that 3*C* inclusions are generated at the interface between the substrate and epitaxial layer as a result of substrate surface damage, which can be effectively removed by *in situ* etching [17,18].

**Figure 4:**
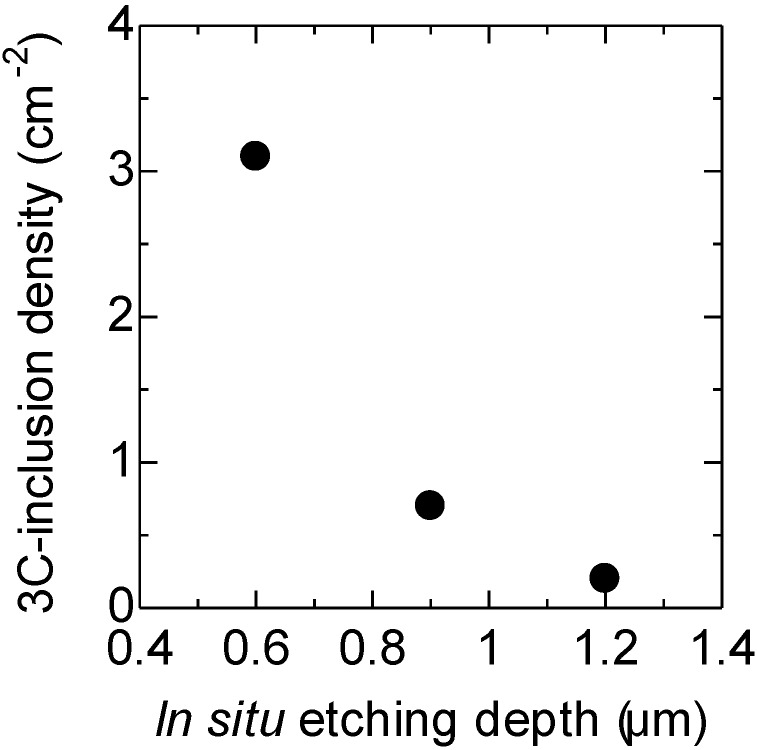
Relationship between 3*C*-inclusion density and *in situ* etching depth for a C/Si ratio of 2.0.

Figure 4 shows the relationship between the 3*C*-inclusion density and *in situ* etching depth. Epitaxial layers were grown at a C/Si ratio of 2.0, because the background carrier concentration was less than 5 × 10^13^ cm^–3^ under these conditions, which is lower by two orders of magnitude than that obtained at the C/Si ratio of 0.7 [6]. The *in situ* etching depth was changed from 0.6 μm to 1.2 μm by varying the *in situ* etching time from 0 min to 40 min. The etching time of 0 min means that the etching was carried out only as the temperature was increased for growth. The 3*C*-inclusion density decreases from 3.1 cm^–2^ to 0.2 cm^–2^ with increasing *in situ* etching depth from 0.6 μm to 1.2 μm, as shown in Figure 4. Thus, *in situ* etching is effective for suppressing 3*C*-inclusion formations, but a depth of over 1.2 μm is needed. It is thought that 3*C*-SiC nucleation sites such as substrate damage sites created by polishing are removed by *in situ* etching.

**Figure 5:**
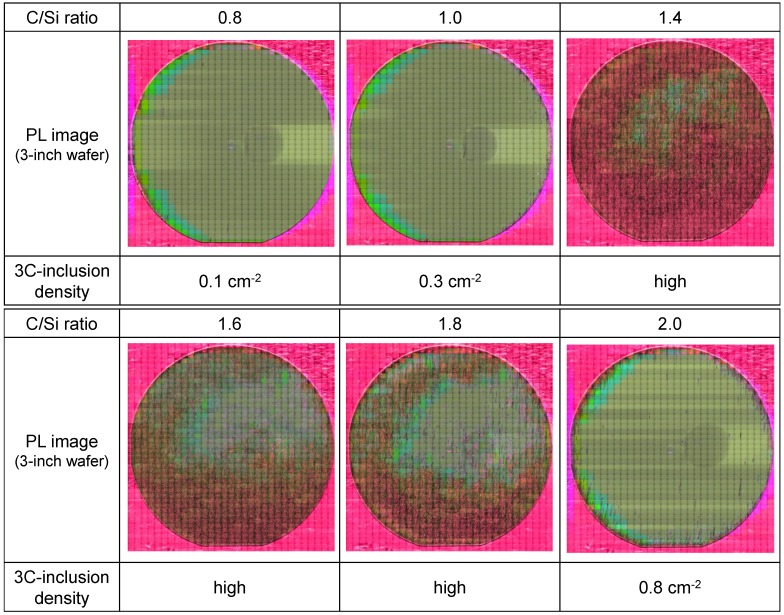
PL images and 3*C*-inclusion densities of epitaxial layers grown at various C/Si ratios with *in situ* etching to a depth of 0.9 μm.

**Figure 6:**
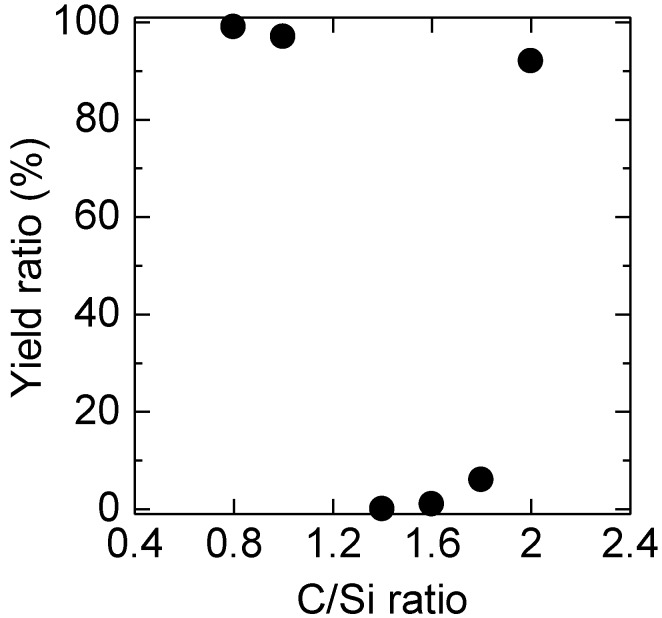
Relationship between yield and C/Si ratio obtained from the PL images shown in Figure 5.

3*C* inclusions were generated both at the interface between the substrate and epitaxial layer, and during epitaxial growth, as described in Section 2.1. Therefore, substrate surface damage is not the only cause of 3*C*-inclusion formations. To examine the other causes of 3*C* inclusions, we investigated the relationship between the 3*C*-inclusion density and the C/Si ratio, because the 3*C*-inclusion density is likely to be significantly affected by the C/Si ratio on the basis of past reports [5,6,19]. Figure 5 shows PL images and the 3*C*-inclusion densities of epitaxial layers grown at various C/Si ratios for *in situ* etching down to a depth of 0.9 μm. In this PL measurement, the incident light was from an Hg-lamp through a band pass filter between 295 nm and 370 nm, and a color CCD was used as the detector. The area without 3*C* inclusions looks green because substrates emit green luminescence presumably due to nitrogen-related levels of 4*H*-SiC. In contrast, areas with 3*C* inclusions look reddish due to their surface roughness. This is because the red wavelength region of the incident light is unintentionally transmitted through the band pass filter because of its imperfection though the transmitted intensity of red wavelength region is considerably lower than that of wavelength between 295 nm and 370 nm. Then, the incident light was scattered by 3C inclusions, and these red wavelength regions were detected by the color CCD. The 3*C*-inclusion densities obtained at C/Si ratios of 0.8, 1.0 and 2.0 are less than 1.0 cm^–2^. In contrast, most part of these PL images obtained at C/Si ratios of 1.4–1.8 looks reddish because the numbers of 3*C*-inclusions were too high, and thus these 3*C*-inclusion densities could not be calculated. Figure 6 shows the relationship between the yield and the C/Si ratio obtained from the PL images shown in Figure 5. The yield was calculated from the number of 2.5 × 2.5 mm^2^ areas without 3*C* inclusions. The yield is over 90% at C/Si ratios of 0.8, 1.0 and 2.0, but is less 10% at C/Si ratios of 1.4–1.8. 3*C*-inclusion formation was suppressed at low C/Si ratios of 0.8 and 1.0 and at a high C/Si ratio of 2.0.

It has been reported that 3*C*-SiC nucleation tends to occur when the terrace width increases [2]. In this experiment, the terrace width measured by AFM was about 500 nm regardless of the C/Si ratio, because step bunching caused by the Schwoebel effect [20] was generated in all samples because of the deep *in situ* etching. It is equally important that surface diffusion length of adatoms increases with decreasing C/Si ratio [21]. At C/Si ratios of 0.8 and 1.0, the diffusion length of adatoms would be long enough for all adatoms to reach steps, and only step-controlled epitaxial growth would occur. As a result, the 3*C*-inclusion density was low. On the other hand, there were presumably many adatoms that could not reach the steps because of the short diffusion length at C/Si ratios of 1.4–2.0. In this situation, both step-controlled growth by the adatoms that did reach the steps and nucleation by the adatoms that did not the reach steps occurred. Next, it is noted that 3*C*-SiC is more stable than 4*H*-SiC at low C/Si ratios; *i.e.*, 4*H*-SiC is more stable than 3*C*-SiC at high C/Si ratio [22,23]. It has been reported that growth at low C/Si ratio introduces many carbon vacancies, which leads to compression of the crystal lattice thus supporting the cubic structure of the layer [22,23]. In contrast, growth at high C/Si ratio reduces carbon vacancies, which is favorable for the hexagonal structure [22,23]. Therefore, step-controlled growth, and 3*C*-SiC nucleation due to the stability of 3*C*-SiC occur when the C/Si ratio is 1.4–1.8. As a result, a high 3*C*-inclusion density was obtained under these conditions. At a C/Si ratio of 2.0, step-controlled growth, and 4*H*-SiC nucleation due to the stability of 4*H*-SiC would occur. As a result, 3*C*-SiC nucleation could be suppressed. 4*H*-SiC nucleation patterns could not be observed, and the reason is not known. Conceivably, 4*H*-SiC nucleation areas may become covered with step-controlled growth. Moreover, other factors such as growth temperature and step height might help for improving the stability of 4*H*-SiC. It has been reported that 4*H*-SiC unseeded sublimation growth occurs at temperature from 1800 °C to 2700 °C [23]. In this study, we grew epitaxial layers at relatively high temperature of 1725 °C. Step heights of the substrates before growth were about 10 nm due to the deep *in situ* etching. It means that 4*H*-SiC stacking widely appeared to the substrate surface, which presumably improved the stability of 4*H*-SiC. Indeed, it has been reported that 4*H*-SiC epitaxial growth on $\left(11\bar{2}0\right)$ substrates improves the 4*H*-SiC stability compared to growth on Si-face substrates [24,25]. Further investigation about the growth mechanism at a high C/Si ratio of 2.0 is needed, but the stability of 4*H*-SiC is certainly important.

We found that 3*C*-inclusion formation could be suppressed at high C/Si ratios by removing substrate surface with deep *in situ* etching and because of the stability of 4*H*-SiC at high C/Si ratios.

### 2.3. Post-Processing for 4H-SiC Si-Face Epitaxial Layers with a 1° Off-Angle

**Figure 7:**
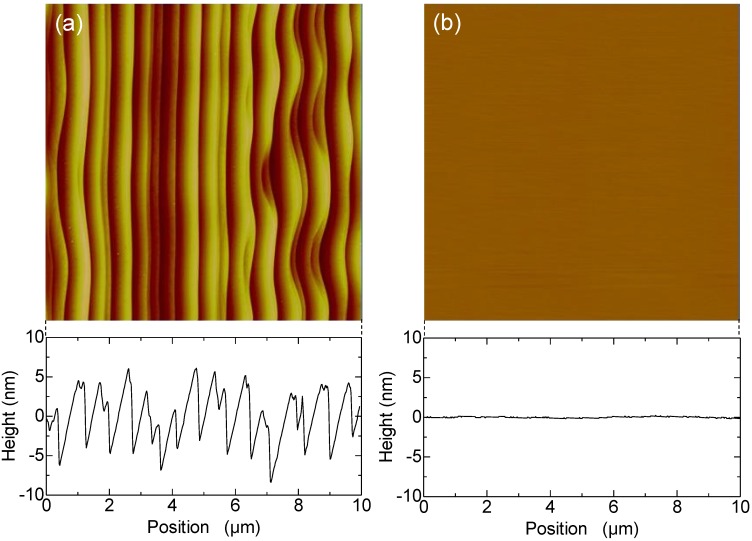
AFM images and the cross-sectional profiles of (**a**) as-grown and (**b**) chemical mechanical polishing (CMP)-treated epitaxial layers grown at a C/Si ratio of 2.0 with *in situ* etching to a depth of 1.2 μm.

The surfaces of the epitaxial layers grown in this experiment should be planarized by post-processing, because the as-grown layers are rough due to step bunching. Figure 7a,b shows the AFM images and cross-sectional profiles of as-grown and chemical mechanical polishing (CMP)-treated epitaxial layers, respectively, grown at a C/Si ratio of 2.0 with *in situ* etching to a depth of 1.2 μm. The as-grown epitaxial layer has a rough surface because of step bunching generated by the deep *in situ* etching, but the rough surface becomes smooth after the CMP treatment. It has been reported that a CMP-treated epitaxial layer with a 4° off-angle shows better oxide reliability than an as-grown one; *i.e.*, epitaxial-surface planarization by CMP treatment is effective for improving the oxide reliability [26]. It is obvious that the epitaxial layers with 1° off-angles grown in this experiment can be applied to devices after CMP treatment.

## 3. Experimental Section

Epitaxial growth was performed using a horizontal hot-wall CVD system. The experimental setup is described in Reference [14] in detail. After CMP treatment, 3- or 4-inch conventional n-type 4*H*-SiC Si-face wafers with a 1° off-angle toward the $<11\bar{2}0>$ direction were used as substrates.

The *in situ* etching was performed in an H_2_ atmosphere. The temperature, pressure, and H_2_ flow rate used for the *in situ* etching were 1725 °C, 6.3 kPa, and 100–134 slm, respectively. The growth proceeded for 2 h at the same temperature, pressure, and H_2_ flow rate as used for the *in situ* etching. The SiH_4_ (10% in H_2_) flow rate was 500–600 sccm, and the C_3_H_8_ (10% in H_2_) flow rate was varied to change the C/Si ratio. The growth rate was 4.0–4.8 μm/h.

The *in situ* etching depth was estimated from the etching rate of the epitaxial layers, because it is difficult to directly measure the etching depth of the substrates. The thickness was investigated by using conventional Fourier transform infrared spectroscopy and taking an average of 137 points.

The surface morphology of the epitaxial layers was observed using tapping-mode AFM. The PL images and PL spectrum were obtained from PL measurements under excitation by an Hg-lamp through a band pass filter between 295 nm and 370 nm and by an He-Cd laser (325 nm), respectively, at room temperature.

## 4. Conclusions 

We grew epitaxial layers on 4*H*-SiC Si-face substrates with 1° off-angles and investigated the 3*C* inclusions with the goal of suppressing the formation of these defects even for growth at a high C/Si ratio. It is thought that the trigger of 3*C*-inclusion formation is 3*C*-SiC nucleation. We succeeded in suppressing 3*C* inclusion formation by performing deep *in situ* etching and using a high C/Si ratio. 3*C*-inclusion formation could be suppressed by removing the substrate surface damage by deep *in situ* etching and because of the 4*H*-SiC stability at high C/Si ratios.

## References

[1] Feng G., Suda J., Kimoto T. (2009). Characterization of major in-grown stacking faults in 4*H*-SiC epilayers. Physica B.

[2] Kimoto T., Matsunami H. (1994). Surface kinetics of adatoms in vapor phase epitaxial growth of SiC on 6H SiC{0001} vicinal surfaces. J. Appl. Phys..

[3] Kimoto T., Itoh A., Matsunami H. (1997). Step-Controlled epitaxial growth of high-quality SiC layers. Phys. Stat. Sol. B.

[4] Tamura K., Kudou C., Masumoto K., Nishio J., Kojima K. (2014). Homo-epitaxial growth on 2° off-cut 4H-SiC(0001) Si-face substrates using H_2_-SiH_4_-C_3_H_8_ CVD system. Mater. Sci. Forum.

[5] Kojima K., Masumoto K., Ito S., Nagata A., Okumura H. (2013). 4H-SiC homoepitaxial growth on substrate with vicinal off-angle lower than 1°. ECS J. Sol. State Sci. Tech..

[6] Masumoto K., Kojima K., Okumura H. (2013). The growth of 3-inch 4H-SiC Si-face epitaxial wafer with vicinal off-angle. Mater. Sci. Forum.

[7] Harada S., Ito S., Kato M., Takatsuka A., Kojima K., Fukuda K., Okumura H. (2010). Isotropic channel mobility in UMOSFETs on 4H-SiC C-face with vicinal off-angle. Mater. Sci. Forum.

[8] Masumoto K., Ito S., Goto H., Yamaguchi H., Tamura K., Kudou C., Nishio J., Kojima K., Ohno T., Okumura H. (2014). Conversion of basal plane dislocations to threading edge dislocations in growth of epitaxial layers on 4H-SiC substrates with a vicinal off-angle. Mater. Sci. Forum.

[9] Kosciewicz K., Strupinski W., Teklinska D., Mazur K., Tokarczyk M., Kowalski G., Olszyna A. (2011). Epitaxial growth on 4H-SiC on axis, 0.5°, 1.25°, 2°, 4°, 8° off-axis substrates-defects analysis and reduction. Mater. Sci. Forum.

[10] Skowronski M., Ha S. (2006). Degradation of hexagonal silicon-carbide-based bipolar devices. J. Appl. Phys..

[11] Ohno T., Yamaguchi H., Kuroda S., Kojima K., Suzuki T., Arai K. (2004). Influence of growth conditions on basal plane dislocation in 4H-SiC epitaxial layer. J. Cryst. Growth.

[12] Burk A.A., Tsvetkov D., Barnhardt D., O’Loughlin M.J., Garrett L., Towner P., Seaman J., Deyneka E., Khlebnikov Y., Palmour J.W. (2012). SiC epitaxial layer growth in a 6 × 150 mm warm-wall planetary reactor. Mater. Sci. Forum.

[13] Miyasaka A., Norimatsu J., Fukada K., Tajima Y., Muto D., Kimura Y., Odawara M., Okano T., Momose K., Osawa Y. (2013). Step-bunching free and 30 μm-thick SiC epitaxial layer growth on 150 mm SiC substrate. Mater. Sci. Forum.

[14] Masumoto K., Kudou C., Tamura K., Nishio J., Ito S., Kojima K., Ohno T., Okumura H. (2013). Growth of silicon carbide epitaxial layers on 150-mm-diameter wafers using a hot-wall chemical vapor deposition. J. Cryst. Growth.

[15] Larkin D.J. (1997). SiC dopant incorporation control using site-competition CVD. Phys. Stat. Sol. B.

[16] Mahadik N.A., Stahlbush R.E., Qadri S.B., Glembocki O.J., Alexson D.A., Hobart K.D., Caldwell J.D., Myers-Ward R.L., Tedesco J.L., Eddy C.R. (2011). Structure and morphology of inclusions in 4° offcut 4H-SiC epitaxial layers. J. Electron. Mater..

[17] Hassan J., Bergman J.P., Palisaitis J., Henry A., McNally P.J., Anderson S., Janzen E. (2010). Growth and properties of SiC on-axis homoepitaxial layers. Mater. Sci. Forum.

[18] Powell J.A., Petit J.B., Edgar J.H., Jenkins I.G., Matus L.G., Yang J.W., Pirouz P., Choyke W.J., Clemen L., Yoganathan M. (1991). Controlled growth of 3CSiC and 6HSiC films on lowtiltangle vicinal (0001) 6HSiC wafers. Appl. Phys. Lett..

[19] Nakamura S., Kimoto T., Matsunami H. (2003). Homoepitaxy of 6H-SiC on nearly on-axis (0001) faces by chemical vapor deposition Part I: Effect of C/Si ratio on wide-area homoepitaxy without 3C-SiC inclusions. J. Cryst. Growth.

[20] Ishida Y., Takahashi T., Okumura H., Arai K., Yoshida S. (2009). Origin of giant step bunching on 4H-SiC (0001) surfaces. Mater. Sci. Forum.

[21] Kimoto T., Matsunami H. (1995). Surface diffusion lengths of adatoms on 6HSiC{0001} faces in chemical vapor deposition of SiC. J. Appl. Phys..

[22] Tairov Y.M., Tsvetkov V.F. (1983). Progress in controlling the growth of polytypic crystals. Prog. Cryst. Growth Charact..

[23] Yakimova R., Vasiliauskas R., Eriksson J., Syvajarvi M. (2012). Progress in 3C-SiC growth and novel applications. Mater. Sci. Forum.

[24] Bishop S.M., Reynolds C.L., Liliental-Weber Z., Uprety Y., Zhu J., Wang D., Park M., Molstad J.C., Barnhardt D.E., Shrivastava A. (2007). Polytype stability and microstructural characterization of silicon carbide epitaxial films grown on
$\left[11\bar{2}0\right]$- and [0001]-oriented silicon carbide substrates. J. Electron. Mater..

[25] Bishop S.M., Reynolds C.L., Liliental-Weber Z., Uprety Y., Ebert C.W., Stevie F.A., Park J.S., Davis R.F. (2008). Sublimation growth of an *in-situ*-deposited layer in SiC chemical vapor deposition on 4H-SiC$\left(11\bar{2}0\right)$. J. Cryst. Growth.

[26] Yamada K., Ishiyama O., Tamura K., Yamashita T., Shimozato A., Kato T., Senzaki J., Matsuhata H., Kitabatake M. (2014). Reliability of gate oxides on 4H-SiC epitaxial surface planarized by CMP treatment. Mater. Sci. Forum.

